# Reducing the Competition: A Dual-Purpose Ionic Liquid for the Extraction of Gallium from Iron Chloride Solutions

**DOI:** 10.3390/molecules25184047

**Published:** 2020-09-04

**Authors:** Luke M. M. Kinsman, Carole A. Morrison, Bryne T. Ngwenya, Jason B. Love

**Affiliations:** 1 EaStCHEM School of Chemistry, University of Edinburgh, Edinburgh EH9 3FJ, UK; L.M.M.Kinsman@sms.ed.ac.uk (L.M.M.K.); Carole.Morrison@ed.ac.uk (C.A.M.); 2 School of Geosciences, University of Edinburgh, Edinburgh EH9 3FE, UK; Bryne.Ngwenya@ed.ac.uk

**Keywords:** ionic liquid, solvent extraction, sustainability, secondary resources, recycling, NMR spectroscopy, UV-Vis spectrophotometry

## Abstract

The separation of gallium from iron by solvent extraction from chloride media is challenging because the anionic chloridometalates, FeCl_4_^−^ and GaCl_4_^−^, display similar chemical properties. However, we report here that the selective separation of gallium from iron in HCl solution can be achieved using the dual-purpose ionic liquid methyltrioctylammonium iodide in a solvent extraction process. In this case, the reduction of Fe^3+^ to Fe^2+^ by the iodide counterion was found to inhibit Fe transport, facilitating quantitative Ga extraction by the ionic liquid with minimal Fe extraction from 2 M HCl.

## 1. Introduction

Gallium is an important component in materials used in modern electronic devices such as light-emitting diodes (LEDs) and solar panels and is also exploited in biomedical, pharmaceutical, and radiopharmaceutical applications, owing to the similar chemical properties of Ga^3+^ and Fe^3+^ cations [1,2]. There are no abundant natural sources of gallium; instead, it is primarily extracted as a by-product of bauxite and zinc ore processing [3,4]. Due to its limited supply in nature, it is considered a critical element, and so its recovery from alternative sources such as coal fly-ash, mine tailings, or electronic waste is important [3,5,6]. In these cases, however, the presence of iron poses challenging selectivity issues in its separation, for example, by solvent extraction.

Ionic liquids (ILs) are an increasingly established class of extractant that are used either neat or diluted in a hydrophobic solvent to extract various metal ions from aqueous solutions [7,8]. ILs such as trioctylammonium chloride ([TOAH][Cl]) and methyltrioctylammonium chloride ([MTOA][Cl]) have been widely reported as reagents for the recovery of gallium and iron by solvent extraction [9,10,11]. Phase transport is achieved through the formation of charge-neutral supramolecular assemblies such as [MTOA][GaCl_4_], with GaCl_4_^−^ formed under high chloride conditions in the aqueous phase. Most industrial solvent extraction processes operate under chloride, sulfate, or nitrate conditions [7]. As such, solvent extraction processes that feature metalate transport largely exploit chloride media to generate chloridometalates, although processes using other aqueous halides as counterions for ILs have been reported [12]. ILs have also been used in the direct recovery of metals from secondary sources by selective metal dissolution [13]. In this case, a trihalide IL provided an oxidizing agent to dissolve the metal and a cation or additional complexing agent. However, current approaches using ILs do not address the challenges in selectivity for Fe and Ga. Under high chloride concentrations, both of these metals exist as the tetrahedral metalates, [FeCl_4_]^−^ and [GaCl_4_]^−^, for which current outer-sphere, cationic receptors do not discriminate [1,2].

Recently, the selective recovery of Ga from iron mine tailings was reported [14]. Leaching of the metals using 8 M HCl generated a mixture of Ga and Fe chlorides which were separated by reducing Fe^3+^ to Fe^2+^ using SnCl_2_. The iron was not extracted easily at this oxidation state, whereas the monoanion [GaCl_4_]^−^ was extracted with tributylphosphate (TBP, 10% in benzene), albeit with 20–30% co-extraction of iron.

In this work, we report a novel combination of the selective reduction of Fe^3+^ and recovery of Ga^3+^ by solvent extraction using the dual-purpose IL methyltrioctylammonium iodide ([MTOA][I]). Mass spectrometry, NMR spectroscopy, and UV-Vis spectrophotometry confirm that the iodide functions as a reducing agent for Fe^3+^ and that the hydrophobic quaternary ammonium group forms a stable ion pair with [GaCl_4_]^−^ in the organic phase, facilitating phase transport and separation in one step. 

## 2. Results and Discussion

**Figure 1:**
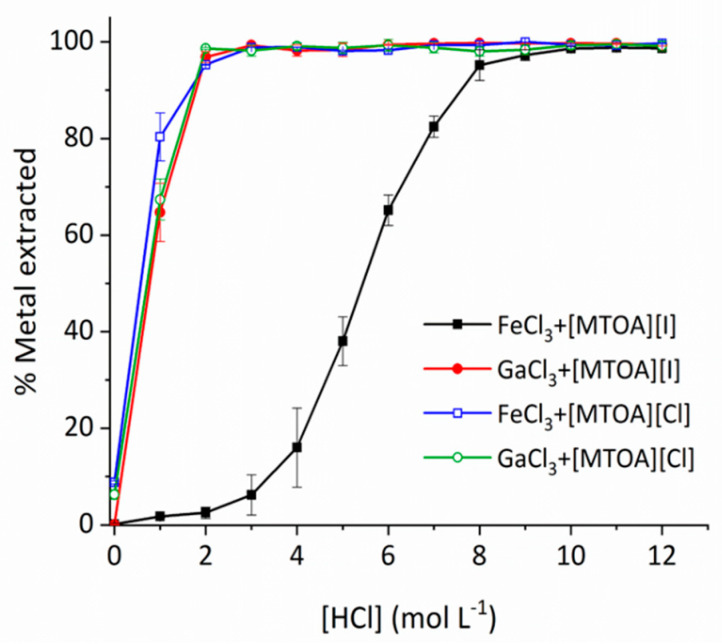
Percentage of gallium and iron extracted by [MTOA][I] at varying [HCl]. Interpolation used to aid the eye only. Experiments were performed in duplicate, and the results are reported as an average.

The transport of gallium into a toluene solution of the iodide IL [MTOA][I] from a binary equimolar mixture of 0.01 M FeCl_3_ and GaCl_3_ in varying concentrations of hydrochloric acid solutions was tested and shows excellent selectivity for gallium between 1 and 4 M HCl (Figure 1). The amount of iron extracted increases markedly as the concentration of HCl increases above 3 M, likely due to a greater proportion of Fe existing as [FeCl_4_]^−^ in solution. In contrast, similar experiments under the same conditions using the chloride IL [MTOA][Cl] show that both iron and gallium are efficiently extracted at concentrations greater than 1 M HCl (Figure 1). Gallium is readily stripped from the organic phase by a fresh aqueous phase of water, whereas <5% is stripped using 2 M HCl.

**Figure 2:**
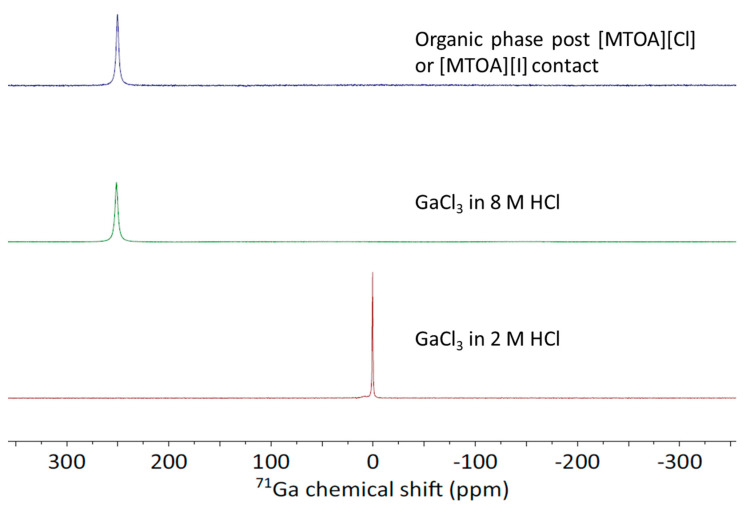
Top: ^71^Ga NMR spectrum of 0.1 M [MTOA][I] or [MTOA][Cl] in toluene after contact with 0.01 M GaCl_3_ dissolved in 2 M HCl. Middle: ^71^Ga NMR spectrum of 0.01 M GaCl_3_ dissolved in 8 M HCl. Bottom: ^71^Ga NMR spectrum of 0.01 M GaCl_3_ dissolved in 2 M HCl.

The nature of the gallium species extracted under these conditions was probed by ^71^Ga NMR spectroscopy (Figure 2). After contact of a GaCl_3_ solution in 2 M HCl with either 0.1 M [MTOA][Cl] or [MTOA][I] in toluene, a single resonance is seen at 250 ppm for both organic phases, consistent with the formation of the [GaCl_4_]^−^ anion [15]. In contrast, the ^71^Ga NMR spectra of aqueous solutions of GaCl_3_ in 0 to 7 M HCl show a peak at 0.0 ppm assigned to the hexahydrate [Ga(H_2_O)_6_][Cl_3_] [15]. The metalate, [GaCl_4_]^−^, is only observed in aqueous solutions above 8 M HCl, upon which the ^71^Ga resonance shifts to 250 ppm. As the metalate is not initially present at 2 M HCl, it is likely that formation of the chlorogallate [GaCl_4_]^−^ occurs at the interface between the two phases, which favors the assembly of a stable ion pair with the quaternary ammonium cation in the organic phase. 

Electrospray ionization mass spectrometry (ESI-MS) was also used to probe the organic phase speciation of extracted Ga and Fe solutions (Supplementary Materials Figures S1 and S2). Organic phases resulting from extractions from 2 M HCl show no evidence of mixed halometalates such as [GaCl_3_I]^−^ or [FeCl_3_I]^−^, and instead the anions [FeCl_4_]^–^ and [GaCl_4_]^−^ are seen in the negative-ion spectrum, while ([MTOA]_2_[GaCl_4_])^+^ is one of the dominant molecular ions in the positive-ion spectrum. The absence of anions such as [GaCl_3_I]^−^ in the negative-ion mass spectrum suggests that [GaCl_4_]^−^ is initially formed in the aqueous phase (or at the interface) prior to transport across to the organic phase, as opposed to the transport of the neutral complex GaCl_3_ with subsequent metalate formation in the organic phase due to the presence of I^−^ ions. 

**Figure 3:**
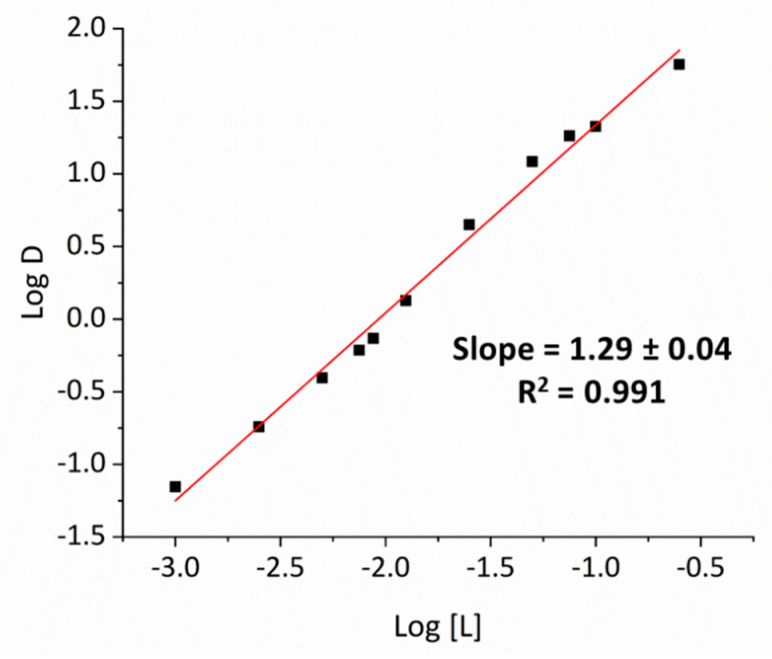
Slope analysis for the transport of Ga by [MTOA][I]. Conditions: GaCl_3_ (0.01 M) in HCl (2.0 M, 2 mL), contacted with [MTOA][I] (0.001 to 0.25 M) in toluene (2 mL) for 1 h at RT with magnetic stirring.

Slope analysis (Log D vs. Log [L]) of the extracted Ga species analyzed for extractant concentrations of 0.001 to 0.25 M at 2 M HCl gives an L:Ga ratio of approximately 1 (Figure 3) and suggests that the simple ion pair [MTOA][GaCl_4_] is present in the organic phase. In addition, there is no correlation between Ga transport and [H^+^] concentration from slope analysis (Log D vs. Log [H^+^]) of varying [H^+^] concentrations of 0.01 to 1 M at 2 M NaCl (Figure S3), ruling out transport of neutral species such as HGaCl_4_ and therefore confirming an ion-exchange extraction mechanism between I^−^ and [GaCl_4_]^−^.

**Figure 4:**
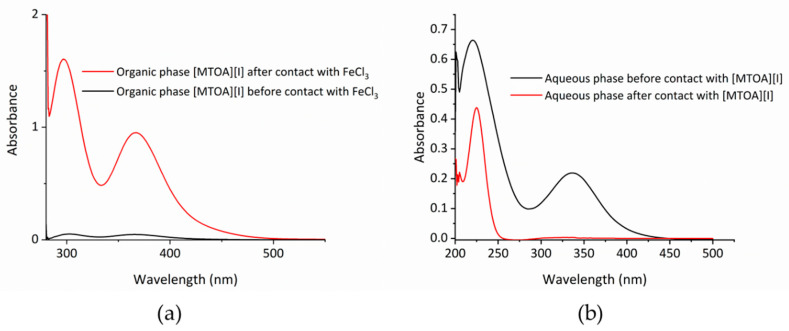
UV-Vis spectra. (**a**) Toluene solutions of [MTOA][I] before (black) and after (red) contact with FeCl_3_. Each solution diluted 500× in toluene. (**b**) The 2 M HCl solutions of FeCl_3_ before (black) and after (red) contact with 0.1 M [MTOA][I] in toluene. Each solution diluted 100× in 2 M HCl.

Evidence for reduction of Fe^3+^ by I^−^ during extractions is apparent, as the color of the organic phase changes from bright yellow to deep red, and the initially yellow aqueous phase turns colorless. The deep red color of the organic phase is consistent with the presence of the triiodide anion, I_3_^−^, and is supported by the appearance of the absorption at 375 nm in the UV-Vis spectrum of the metal-loaded organic phase (Figure 4a) [16]. The UV-Vis spectrum of the Fe^3+^ aqueous phase at 2 M HCl prior to contact with [MTOA][I] shows two absorption maxima at 220 and 336 nm which are consistent with the presence of FeCl^2+^ (Figure 4b) [17,18]. After contact with [MTOA][I], the UV-Vis spectrum of the aqueous phase bleaches and shows only one absorption maximum at 225 nm due to the presence of aqueous Fe^2+^ (Figure 4b); Fe(II) chloridometalates are unlikely to be present, as high chloride concentrations are needed for their formation, and therefore no extraction of Fe(II) complexes is seen using the anion extractant [MTOA][I] [19,20].

## 3. Materials and Methods 

Unless otherwise stated, all solvents and reagents were purchased from Sigma-Aldrich, Fisher Scientific UK, Alfa Aesar (Heysham, UK), Acros Organics (Geel, Belgium), or VWR International (Lutterworth, UK) and used without further purification. Deionized water was produced using a Milli-Q purification system. ^1^H and ^13^C NMR Spectra were recorded on a Bruker AVA500 spectrometer operating at 500.12 and 125.76 MHz for ^1^H and ^13^C, respectively. ^71^Ga NMR spectra were recorded on a Bruker PRO500 spectrometer at 152.55 MHz.

### 3.1. General Solvent Extraction Procedure

Aqueous solutions of FeCl_3_ and GaCl_3_ (0.01 M) in 0–12 M HCl (2 mL) were contacted with a toluene organic phase (2 mL) containing either methyltrioctylammonium iodide or methyltrioctylammonium chloride (0.1 M) and stirred (1 h, 1000 rpm, 25 °C). The phases were then separated physically, and samples were from each taken and diluted with 1-methoxy-2-propanol for ICP-OES analysis. Samples from relevant phases were also taken for UV-VIS, NMR, and ESI-MS analysis as required.

### 3.2. Synthesis of Methyltrioctylammonium Iodide ([MTOA][I])

Following a standard preparation [21], iodomethane (4.44 g, 31 mmol) was added dropwise to a stirred solution of trioctylamine (8.85 g, 25 mmol) in THF (100 mL), and the mixture was stirred at 40 °C for 12 h under a flow of N_2_. The crude mixture was concentrated under vacuum to yield a viscous orange oil (100%) and diluted with toluene to form a 1.0 M stock solution.

^1^H NMR (500 MHz, CDCl_3_): δ_H_ 3.41–3.35 (m, 6H, NC**H**_2_), 3.24 (s, 3H, NC**H**_3_), 1.74–1.64 (m, 6H, C**H**_2_), 1.43–1.23 (m, 30H, C**H**_2_), 0.89 (t, *J* = 6.8 Hz, 9H, CH_2_C**H**_3_). ^13^C{^1^H} NMR (126 MHz, CDCl_3_): δ_C_ 61.70, 48.83, 31.62, 29.10, 29.01, 26.30, 22.57, 22.43, 14.04.

## 4. Conclusions

It is clear from this work that the quaternary ammonium salt, [MTOA][I], functions as a novel dual-purpose reductant and extractant, efficiently and selectively separating Ga^3+^ from Fe^3+^ in a single step between 1–4 M HCl by reduction of Fe^3+^ to Fe^2+^ and transport of Ga as its metalate [GaCl_4_]^−^ by anion exchange. This process is operationally simple, featuring low-cost, readily available reagents. Excellent separation of Ga from Fe occurs under low to moderate HCl concentrations and eliminates the need for external reducing agents such as Fe powder or SnCl_2_. Back extraction (stripping) of gallium from the organic phase occurs readily with water. In principle, the organic phase could be regenerated by contact with aqueous potassium iodide and a mild reducing agent, such as sodium thiosulfate [22].

## Supplementary Materials

The following supplementary materials are available online. Figure S1: Negative ion ESI-MS of [MTOA][I] in toluene after contact with FeCl_3_ and GaCl_3_ in 2 M HCl. Figure S2: Positive ion ESI-MS of [MTOA][I] in toluene after contact with FeCl_3_ and GaCl_3_ in 2 M HCl. Figure S3: Slope analysis for the transport of Ga by [MTOA][I] with varying [H^+^].
