# Supplementary material for: Reducing the Competition: A Dual-Purpose Ionic Liquid for the Extraction of Gallium from Iron Chloride Solutions
Source: Molecules. 2020 Sep 4;25(18):4047. doi: 10.3390/molecules25184047 (PMC7570643; doi:10.3390/molecules25184047)
Supplement: Supplementary file 1 [file molecules-25-04047-s001.pdf]

Supplementary information

Reducing the competition: A dual purpose ionic liquid for the extraction of gallium from iron chloride solutions.

Luke M. M. Kinsman,[1] Carole A. Morrison,[1] Bryne T. Ngwenya,[2] and Jason B. Love[1]*

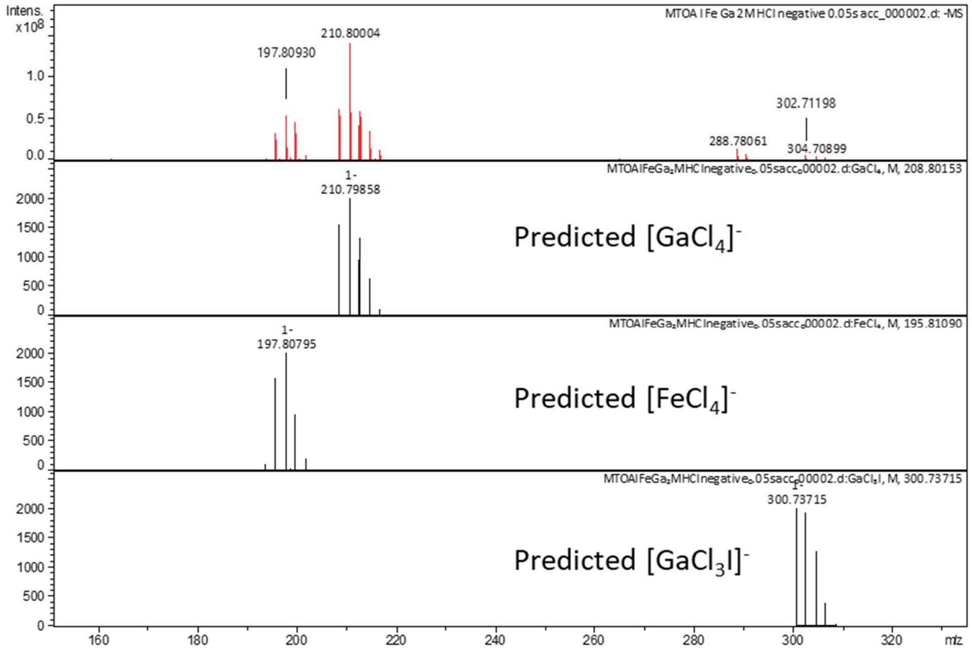

Figure S1. Negative ion ESI-MS of [MTOA][I] in toluene after contact with $FeCl_3$ and $GaCl_3$ in 2 M HCl. Solution diluted in CH_3CN. Real peaks coloured in red and predicted peaks coloured black.

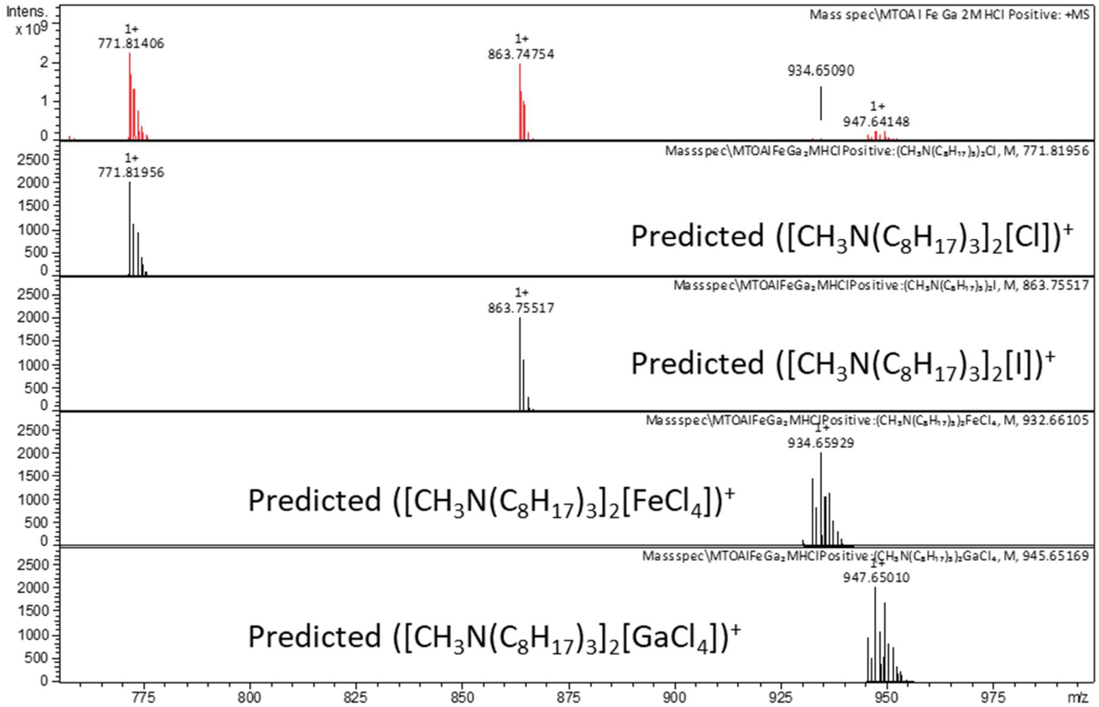

Figure S2. Positive ion ESI-MS of [MTOA][I] in toluene after contact with $FeCl_3$ and $GaCl_3$ in 2 M HCl. Solution diluted in CH_3CN. Real peaks coloured in red and predicted peaks coloured black.

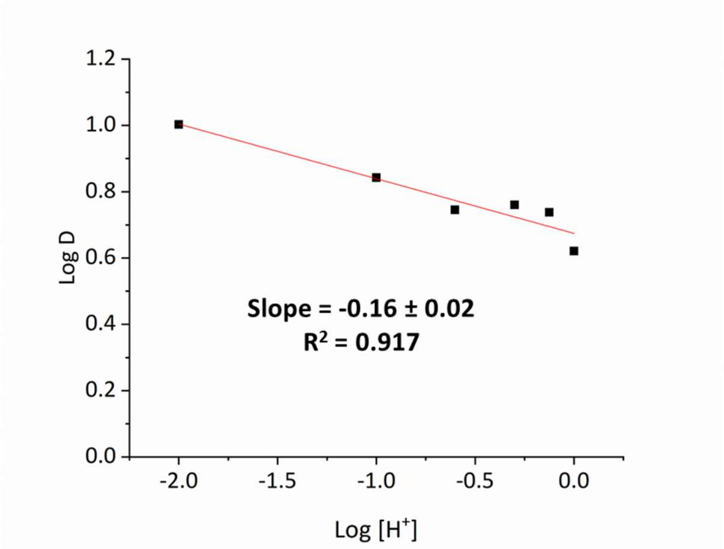

Figure S3. Slope analysis for the transport of Ga by [MTOA][I] with varying [H^+]. Slope of almost zero indicates no relationship between [H^+] and Ga transport, suggesting an ion exchange mechanism instead of transport of $HGaCl_4$. Conditions: $GaCl_3$ (0.01 M) in in 2 M NaCl varying HNO_3 (0.01 M – 1 M, 2 mL), contacted with [MTOA][I] (0.1 M) in toluene (2 mL) for 1 h at RT with magnetic stirring.
